# INTEGRATIVE ANALYSIS OF ALZHEIMER’S DISEASE GWAS TO DEVELOP A NEW POLYGENIC PREDICTOR AND TEST BIOSOCIAL ETIOLOGY

**DOI:** 10.1093/geroni/igz038.1262

**Published:** 2019-11-08

**Authors:** Amal Harrati

**Affiliations:** Stanford School of Medicine, Stanford, California, United States

## Abstract

Alzheimer’s disease (AD) has genetic and environmental causes and etiology is thought to reflect interplay among these factors. A barrier to integration of genetic and environmental etiologic factors in research to inform prevention and intervention is poor understanding of AD genetics beyond APOE4. We used the new Genomic SEM methodology to conduct integrative analysis of results from several AD genome-wide association studies (GWAS), including brain-imaging and autopsy AD GWAS, to derive a novel, polygenic genetic predictor of AD. We applied this polygenic predictor in the US Health and Retirement Study to test associations with (1) cognitive aging measured from repeated-measures longitudinal cognitive-test data; and (2) blood-chemistry-based biological age algorithms. In further exploratory analysis, we tested if risk measured by the novel AD polygenic predictor was correlated with and/or buffered by a known environmental factor influencing AD etiology, life-course socioeconomic position. Results map new directions for biosocial AD research.

